# An Assessment of the Thermal Behavior of Envelope Surface Coatings with Different Colors

**DOI:** 10.3390/polym13010082

**Published:** 2020-12-28

**Authors:** Iwona Pokorska-Silva, Marta Kadela, Marcin Małek, Lidia Fedorowicz

**Affiliations:** 1 Faculty of Civil Engineering, Silesian University of Technology, Akademicka 5 Str., 44-100 Gliwice, Poland; iwona.pokorska-silva@polsl.pl; 2 Building Research Institute (ITB), Filtrowa 1 Str., 00-611 Warsaw, Poland; 3 Faculty of Civil Engineering and Geodesy, Military University of Technology in Warsaw, Gen. Sylwestra Kaliskiego 2 Str., 00-908 Warsaw, Poland; marcin.malek@wat.edu.pl; 4 Faculty of Architecture, Civil Engineering and Applied Arts, Katowice School of Technology, Rolna 43 Str., 40-555 Katowice, Poland; lidiafedorowicz@gmail.com

**Keywords:** polyethene non-woven, PVC cover, building envelope, building surface temperature, absorption, in situ measurement, air temperature, climate date, SEM analysis, surface roughness

## Abstract

Contemporary solar power engineering enables the conceptual interlocking of the shape of a building object with its location, structural design, and external envelope, as well as applied materials. Suitably selected solutions involving the structure, shape, construction, and location of a building can significantly improve the thermal balance of rooms in a building. Particularly valuable and warranted are studies involving various solutions for building partitions contributing to a considerable improvement in the thermal balance of a building. This article presents the results of research on temperature changes on the surface of the external part of a partition coated with layers of different colors. For the lightest coating (white), both the average temperature obtained on the and the maximum temperature obtained on the surface were the lowest. With the darker coatings, these temperatures were both higher. The back analyses that were performed indicated lower and higher absorption coefficients, respectively, for the coating compared with the base value for the red coating. Additionally, it was demonstrated that the average surface roughness (*Ra*) after tests in a natural environment decreased by 12.1% for the base (red) coating. For the grey and white samples, a more than two-fold increase in roughness was reported, of 198.6% and 202.0%, respectively. The SEM analysis indicated material loss and discoloration on the sample surfaces.

## 1. Introduction

In recent years, increasingly more emphasis has been placed on the rational use of energy and waste management in building materials [1,2,3,4]. One of the main issues of energy saving involves heat protection of external building partitions [5]. The most frequently undertaken action to reduce energy consumption is to increase the thermal resistance of external partitions—improving the insulation of partitions by increasing the thickness of the thermal insulation [6]. In this way, attempts to minimize heat loss in rooms are more and more effectively separated from external weather conditions, which can often lead to the deterioration of the microclimate inside the building. Another way to save energy is to improve the existing design and material solutions [7,8,9,10] and to look for alternative solutions, e.g., using renewable energy (such as solar radiation [11,12]; wind, water, geothermal energy [13,14,15]; or biomass). In energy-conscious (solar) building design, attention is paid to many elements—finding solutions regarding the structure, shape, construction, and location of the building, including carefully selected parameters for the partition, facilitating the possibility of using solar radiation energy, which can significantly improve the heat balance of rooms in the building [16,17,18,19]. The potential for using solar radiation energy is enormous, both coincidentally, taking advantage of the laws of physics, and in a planned manner, directly or through the use of appropriate methods and devices, using solar radiation derivatives [12].

An important role in a building’s energy balance is played by energy transport through its envelope [20]. The exchange of heat on the outer surface of the non-transparent external partition (1) takes place by convection and radiation with the immediate surroundings, by radiation from the sky, and also by irradiation of the partition surface with solar radiation [12].

(1) $$-\lambda_{p}\frac{\partial T_{p}\left(t\right)}{\partial x_{p}}|{}_{x=e}=h_{e}\left(t\right)\left[T_{e}\left(t\right)-T_{p,e}\right]+q_{sky}\left(t\right)+q_{s}\left(t\right)$$

where:

*T_p_ = T_p_* (*x*, *y*, *z*, *t*): temperature of partition surface (°C)

*λ_p_*: thermal conductivity index (W·(m·K)^−1^)

*q_sky_* (*t*): heat flux exchanged with the sky (W·m^−2^)

*q_s_* (*t*): flux of absorbed solar radiation energy (W·m^−2^)

*h_e_* (*t*) *= h_cv e_* (*t*) *+ h_n rd e_* (*t*): heat transfer coefficient by convection and radiation from the outside (W·(m^2^·K)^−1^)

Each component of the balance can be determined separately with accuracy.

The flux of the energy absorbed by the external surface is described by Equation (2), as follows:

(2) $$q_{s}\left(t\right)=G_{s}\left(t,\beta ,\gamma \right)\alpha$$

where:

*G_s_*: solar radiation intensity (W)

*β*: inclination angle of a given partition

*α*: absorbance of the partition surface

*γ*: azimuth angle

The heat flux passing through the partitions is determined by the temperature values obtained on the surfaces of the partition. The temperatures of the isolated partition surfaces are generally much higher than the temperature of the surrounding outside air. The type of surface and the material of the layer in contact with the external environment are very important [16]. The analyses and measurements of the partition parameters, which have an impact on the partition’s ability to absorb solar radiation, are presented, among others, in the literature [16,17,19,21,22]. Orzechowski and Ziętala [16] presented the measurement methodology of absorbed energy and the measurement results of surfaces covered with white paint and colored with a green dye of different intensities. Grudzińska [17] prepared a mathematical model of solar transmission through a surface layer, and identified the optimal properties of the surface components, allowing for maximum solar gains in the winter and protection from overheating in summer. She indicated that for such a layout of the wall, attempts to ensure maximum radiation gains in the winter and minimum radiation gains in the summer are opposing tasks. Synnefa et al. [21] presented the measured spectral properties of solar radiation and the thermal parameters of ten cool colored coatings. They found that all cool colored coatings containing infrared reflective pigments allowed for obtaining lower surface temperatures than those obtained by means of conventional pigments of the matched color coating. The significance analysis involving the impact of the physical properties of the materials of the individual layers of the partition on the surface temperature of the external partition are presented in [22]. Here, based on the results of the numerical analyses of the temperature changes in the external surfaces, depending on the material parameters of the partition surface, i.e., the absorption coefficient of solar radiation of the partition surface, a considerably large influence on the simulation results was demonstrated for the absorption coefficient characterizing the material of the external layer. In the literature, a numerical simulation was carried out for dome-shaped buildings [23] and its envelope with polyethene (PE) non-woven fabric coated with polyvinylchloride (PVC). Monolithic buildings of an elliptical–spherical shape were erected using the technology patented by the South brothers in 1979 in the United States. The dome, as a fragment of a sphere or rotational ellipsoid, was already considered to be an ideal shape in ancient times. Nowadays, this shape responds to the growing interest in creative and organic shapes in modern architecture [24,25]. Computer-aided design programs now offer new concepts of space that go beyond the Cartesian understanding of form and structure [24,26]. In addition, it fits the trend of looking for an optimal shape for energy efficiency, uses ecology materials and production methods, and has the same strictly controlled internal environment [9,27,28,29]. Dome-shaped building objects have various utility functions (homes, cabins, churches [30], schools, stadiums, bulk storages, and various other facilities) [31,32]. Although the structure of such objects is quite unusual, the initially demonstrated benefits in terms of thermal protection are encouraging [22,33]. However, in order to carry out detailed analyses, it is necessary to create a reliable computational model and to obtain input data, including data for an external layer of the envelope (façade material).

Therefore, based on the above, the objective of this study was to assess the impact of the color of the PE non-woven fabric coated with PVC of different colors, used as the external layer of a dome-shaped building partition on temperature distribution on its surface. These data were used to determine the absorption coefficients using back analyses. In addition, the surface roughness of the material of the external partition was analyzed.

## 2. Methodology

### 2.1. Measurement of Surface Temperature

The studies involved the measurement of the surface temperature of polyethene (PE) non-woven fabric coated with polyvinylchloride (PVC) of different colors, used as the external layer of the partition of buildings erected as thin-walled monolithic reinforced concrete domes. The research was conducted from 1 July 2017 to 31 October 2018.

The following measuring equipment was used:

- YSI 44005 thermistors with a measuring range from −40 to +105 °C and measuring accuracy of 0.5 °C, connected to a Geokon 8002 multi-channel recorder (Lebanon, NH, USA) for surface temperature measurement,
- USB st-171 temperature and humidity recorders with a measuring range for temperatures from −40 to +70 °C and humidity from 0 to 100%, and a measurement accuracy of 0.5 °C and a 3% measurement of temperature and air humidity.

**Figure 1:**
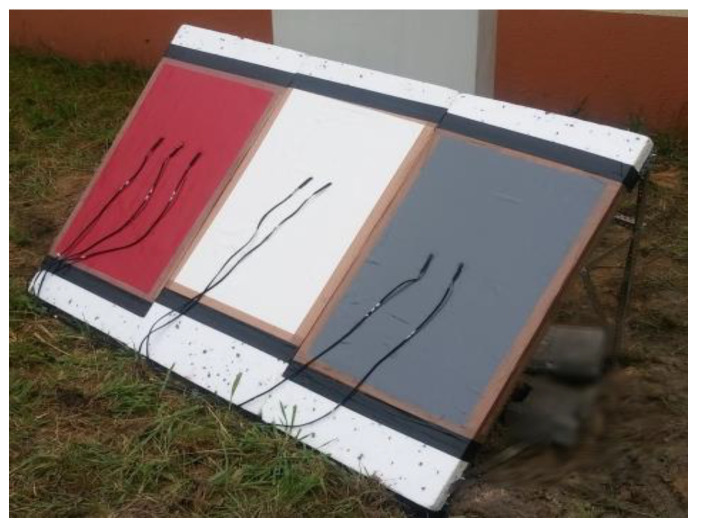
View of the tested partition.

The temperature was measured on three surfaces of different colors (red, white, and grey), as shown in Figure 1. The non-woven fabric was attached to the insulation layer (limiting the way heat flowed from below). The research was carried out by locating the surface in question to the north, inclined at 45° to the horizontal plane. This was adopted, taking into account the measurement capabilities, terrain topology, shading elements, and location of the measurement points for a real building in terms of world direction and slope.

### 2.2. Measurement of Surface Roughness

An Olympus OLS4100 (Tokio, Japan) laser scanning digital non-contact microscope was used to calculate the *Sa* and *Ra* roughness parameters, according to ISO standards (ISO 3274 [34], ISO 4288 [35]). The use of a non-contact method (laser beam) in the measuring procedure for the parameters of the geometric structure of the surface, especially for the roughness profile, significantly improved the measurement accuracy by eliminating the effect of rounding in the measuring tip used in the contact method. A 5× objective lens was applied at 864× total magnification in mixed observation mode. The measurement resolution (laser measurement) was 200 nm. The observed area was 2560–320 µm. The raw *Ra* and *Sa* values were used to obtain data on the bulk surface roughness. *Ra* was measured in ten different areas, with ten profiles chosen from each area approximately equidistant from one another. Five equidistant measurements were taken along the length of the sample and were then rotated by roughly 180 ℃, and a subsequent five additional measurements were taken. The total number of *Ra* and *Sa* measurements were arithmetically averaged to obtain the final values of *Ra* and *Sa*. To verify the robustness of the method itself, reproducibility tests were conducted on five samples.

## 3. Results and Discussion

**Figure 2:**
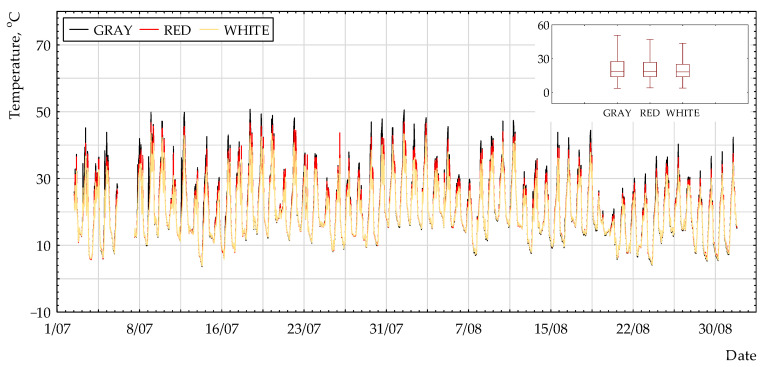
Surface temperature (N_45°) for the period of 1 July to 31 August 2017.

**Figure 3:**
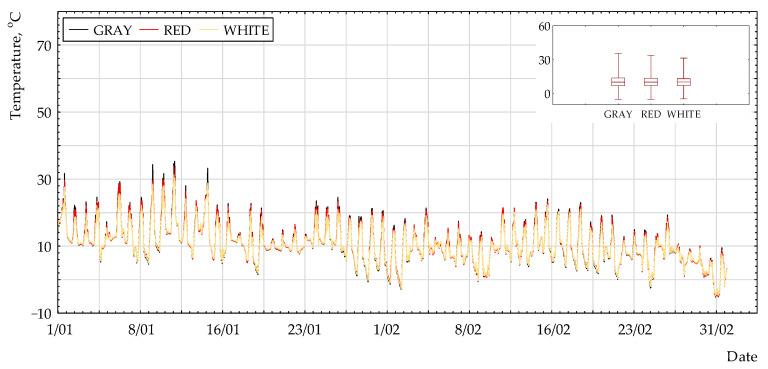
Surface temperature (N_45°) for the period of 1 September to 31 October 2017.

**Figure 4:**
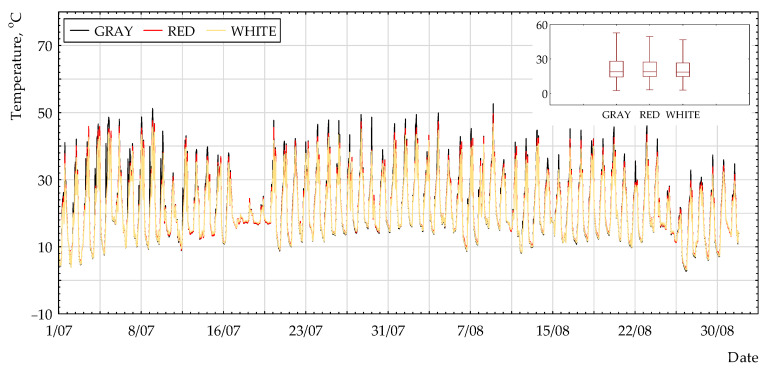
Surface temperature (N_45°) for the period of 1 July to 31 August 2018.

Figure 2, Figure 3 and Figure 4 present the selected results of temperature measurements for the non-woven fabric surface with different colors. It can be observed that the highest values were for grey coating and the lowest for white coating. The difference between all of the coatings was very small.

**Figure 5:**
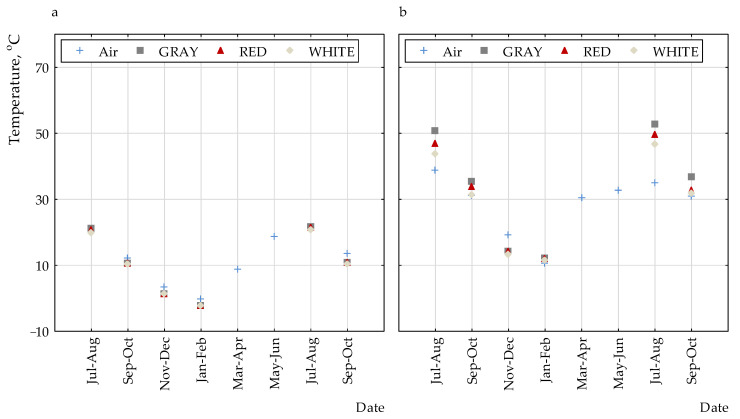
Temperatures of the partition surface for the period of 1 July 2017 to 31 October 2018: (**a**) average and (**b**) maximum.

For measurements of the temperature on the surface of the coatings and the outside air, the average (*T_av_*) and maximum temperatures (*T_max_*) were determined. Table 1 and Figure 5 present the results.

For the lightest coating (white), both the average surface temperature and the maximum temperature were the lowest. With darker coatings, these temperatures were both higher: the average ones for the red surface by 0–0.9 °C and for the grey surface by 0–1.4 °C, and the highest temperatures obtained on the surface: for the red surface by 0.6–3.2 °C and the grey one by 0.8–7.1 °C, relative to the white surface and for a given time interval.

**Figure 6:**
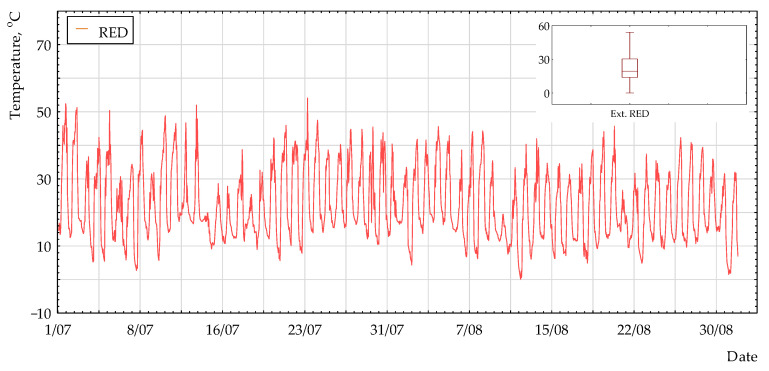
Surface temperature (N_45°) for the period of 1 July to 31 August 2016.

The temperatures of the red coating measured in this study were compared to the surface temperatures of the real building (thin-walled monolithic reinforced concrete dome [33]), whose external walls were covered with the same red coating. Figure 6 presents the temperature measured on the real surface of the building from the north at a place representing a gradient of 45° to the horizon, which was analogous to the measurement in this study. The external walls of a real building were made as layers composed of reinforced concrete class C20/25, 100 mm thick, thermal insulation PUR foam 100 mm thick, and non-woven PE coated with 1 mm-thick PVC in red. The temperature was measured from the north at a place representing a gradient of 45° to the horizon. Table 2 presents a comparison between the temperature results for the envelope tested in this study and the results of temperature measurements for a real building.

**Figure 7:**
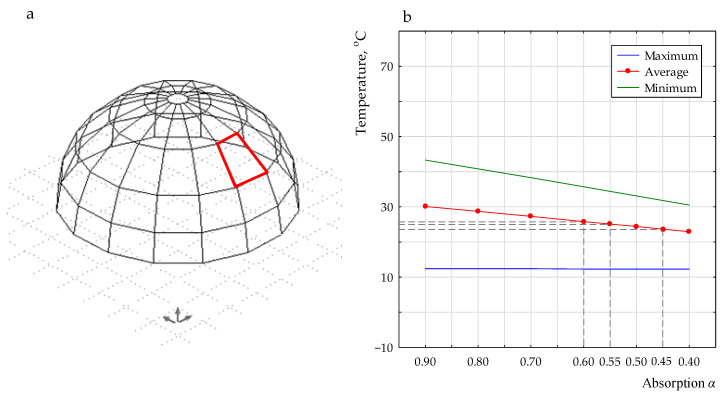
(**a**) Numerical model and (**b**) surface temperature (N_45°) on 5 July obtained in the simulation.

The temperatures obtained for the real partition were higher than temperatures measured in this study. This can be attributed to the fact that the measurements on the partition surface in a real building were carried out at a higher altitude, where solar radiation is more accessible (no disturbances from the immediate surroundings). Because of the lack of a complete real climate base (especially that of solar radiation) and because the measurements were carried out in different time periods, it was not possible to fully compare the results. Therefore, using the previous experience in numerical simulations of thermal process in buildings, the authors followed the same research path, combining in situ tests and the possibility of numerical analysis in order to assess the impact of absorption on the thermal behavior. For this purpose, in the first step, the back analysis for the real building was carried out using the program Environmental Systems Performance. According to the methodology presented in the literature [33], a numerical analysis for a model with an assumed typical metrology year as a boundary condition was performed for 5 July 2016. This model was validated based on the measured temperatures of the external envelope surface of a real building (see the last row in Table 2). The absorption coefficient of solar radiation (α) was assumed to be in range of 0.4 to 0.9 with a step of 0.5. The best fit was obtained for an absorption coefficient equal to 0.55. The correlation between the temperature of the external envelope surface and absorption coefficient, obtained in the numerical analysis, is shown in Figure 7.

The next step was to calculate the percent difference between the temperature of the surface coating with white and grey colors, and the reference (red) coating measured in a field test in Katowice (in this study). For the same percent difference compared with the base values of the surface temperature obtained in the numerical analysis (*T_av_* = 25.1 °C), the absorption coefficients for the white and grey coatings were established from Figure 7. It was determined that the absorption coefficients of solar radiation (α) were 0.60 and 0.45 for the grey and white coatings, respectively. The demonstrated values are in line with the results of other scientists obtained from different materials (see Table 3). It can be observed that α = 0.42 for light concrete and 0.73 for dark concrete, which caused the obtained results in this study to be lower by about 7% and 20% for the white and grey coatings, respectively. However, generally, the radiation absorption of the envelope depends on the incidence angle of sunlight and on the temperature of this envelope, and it is different for different materials and depends on the level of surface treatment of the material (including surface roughness [36]). Rough, dark, and matte surfaces demonstrate the highest absorption. Therefore, in the next step, the surface roughness for the analyzed coating was investigated.

**Figure 8:**
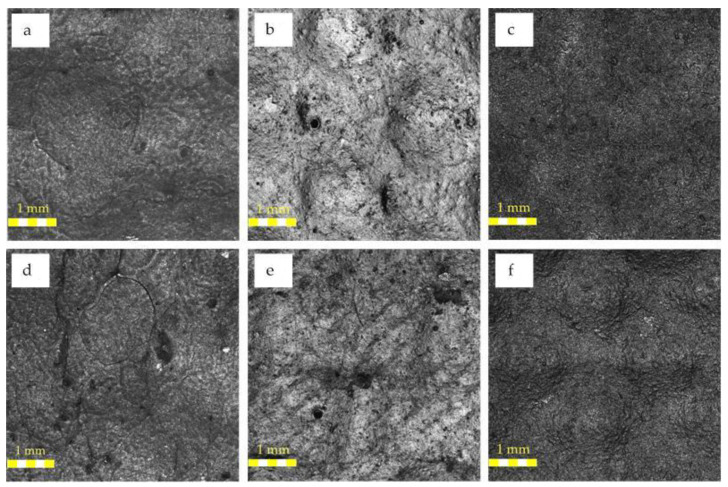
Microstructures of tested samples: reference sample: (**a**) grey, (**b**) red, and (**c**) white. Microstructures of the sample after natural conditions: (**d**) grey, (**e**) red, and (**f**) white.

**Figure 9:**
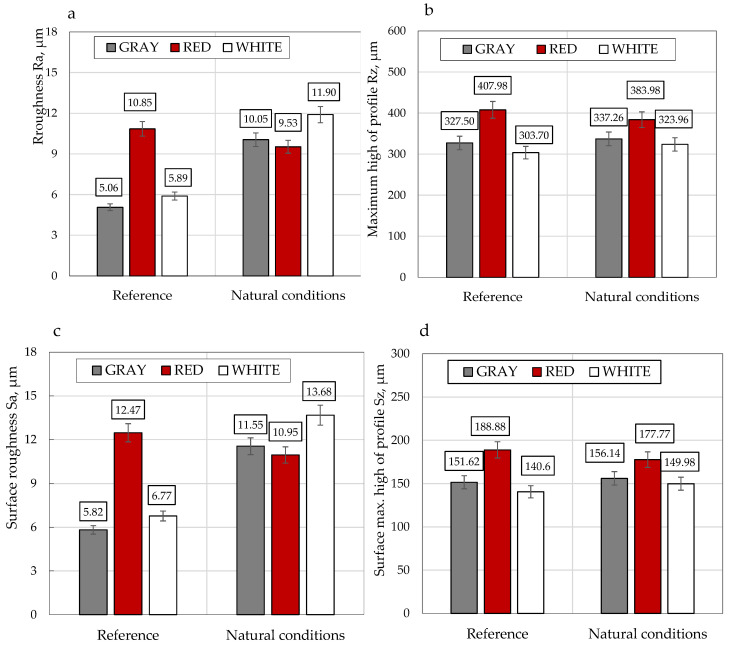
(**a**) Roughness (*Ra*), (**b**) maximum height of the profile (*Rz*), (**c**) surface roughness (*Sa*), and (**d**) surface maximum height of the profile (*Sz*).

The measurement results of the surface roughness for the reference samples (red coating) and the samples exposed to the external climate for 16 months are presented in Figure 8 and Figure 9. Both linear and surface analyses were performed for each of the samples. Because of the immersed PE fabric, the samples of the materials had a non-uniform, corrugated surface. The maximum deviation from the profile for the *Rz* parameter was almost 100 μm. This was achieved by a different, very complex structure for the materials, consisting of many layers of fibers. The “white” and “grey” samples were from the same batch and were characterized by a similar thickness and structure (white, 0.56 mm thick, and grey, 0.58 mm thick). The sample of the red material was thicker and its surface was more corrugated (0.74 mm thick). The *Rz* parameter, after exposure to external conditions, decreased by 5.9% for the red sample, while for the white and grey samples, it increased by 6.7% and 3.0%. The deposition of dirt probably affected the decrease of the *Rz* parameter of the red surface in the material cavities. With respect to the average surface roughness (*Ra*), its values for the grey, white, and red coatings were 5.06 µm, 10.85 µm, and 5.89 µm, respectively. It can be observed that the highest roughness (white coating) was obtained for the lowest absorption coefficient. While for other samples with a similarly obtained absorption coefficient (±0.05), a similar roughness was determined.

After the tests in the natural environment, a slight decrease was also observed for the red sample, of up to 9.53 µm (i.e., 12.1%). However, for the grey and white samples, the roughness more than doubled, up to 10.05 µm (198.6%) and 11.90 µm (202.0%), respectively. On the surfaces of those samples, the highest material loss and discoloration were observed, which is probably connected with the very high impact of solar radiation on these coatings and their degradation. The same effect was observed by I-Ju Kim in [38,39]. He measured surfaces that had been exposed to wear through use as a floor. The values increased with increasing the wear/damage to the surface, which was also observed by the authors. However, the base *Ra* values were slightly lower. The increase in roughness coefficients was caused by the application method. In addition, the surface on which the paints was applied was also uneven, hence the value changes. According to Chen et al. [40], the obtained values were within the range adopted for the materials used—both the values obtained before exposure to sunlight and after exposure. Moreover, Chen observed that after wear, the roughness values almost doubled, which was also observed for the tested samples.

## 4. Conclusions

This paper presents the partition surface temperature variations in the Silesia Region, Poland. The following conclusions can be drawn from the presented results:

The temperatures of the isolated partition surfaces were generally higher than the temperature of the surrounding outside air.

The choice of color for the partition surface evidently affected the temperature distribution obtained on the external surface.

For the lightest coating (white color), both the average surface temperatures and the maximum temperatures were the lowest. With darker coatings, these temperatures were each higher: average temperatures for the red surface by 0–0.9 °C and for the grey surface by 0–1.4 °C, and the highest temperatures obtained on the surface: for the red surface by 0.6–3.2 °C and the grey one by 0.8–7.1 °C, relative to the white surface and for a given time interval.

Based on the back analysis, absorption coefficients of solar radiation (α) of 0.45, 0.55, and 0.60 for white, red, and grey coating were obtained, respectively. In future work, these values should be verified in a laboratory test.

The performed roughness tests demonstrated that the maximum deviation from the profile for the *Rz* parameter was almost 100 μm. The *Rz* parameter, after exposure to external conditions, decreased by 5.9% for the red sample, and increased by 6.7% and 3.0% for the white and grey samples, respectively.

The average surface roughnesses (*Ra*) for the grey, white, and red coating were 5.06 µm, 10.85 µm, and 5.89 µm, respectively. It can be observed that the highest roughness (white coating) was obtained for the lowest absorption coefficient. While for other samples with a similarly obtained absorption coefficient (±0.05), a similar roughness was determined.

The average surface roughness (*Ra*) after the tests in the natural environment also decreased slightly—by 12.1% for the red sample. However, for the grey and white samples, the roughness increased more than twice, up to 198.6% and 202.0%, respectively. The SEM analysis showed material loss and discoloration on the surface of the samples.

The presented results are a part of the research program for coatings used as an external layer for the envelope. Thus, as part of further research, e.g., measurement of the solar radiation and light reflectivity, thermal emissivity of the surface will be carried out, with particular emphasis on these properties after fatigue tests. These results will be used to define a thermal material model that will be used as part of the global real building transformation to a numerical building model.

## Figures and Tables

**Table 1:** Average and maximum surface temperatures of the partition for the period of 1 July 2017–31 October 2018.

		July–August *		September–October		November–December		January–February		March–April *		May–June *		July–August		September–October	
		T_av_	T_max_	T_av_	T_max_	T_av_	T_max_	T_av_	T_max_	T_av_	T_max_	T_av_	T_max_	T_av_	T_max_	T_av_	T_max_
		°C															
Partition	Gray	21.1	50.8	10.6	35.4	1.2	14.2	−2.4	12.2	-	-	-	-	21.7	52.7	10.8	36.6
	Red	20.6	46.9	10.5	33.8	1.2	14.1	−2.3	12.0	-	-	-	-	21.3	49.6	10.8	32.6
	White	19.7	43.7	10.3	31.3	1.2	13.3	−2.2	11.4	-	-	-	-	20.8	46.7	10.4	31.8
Air		21.1	38.8	12.1	31.1	3.4	19.2	−0.3	10.6	8.8	30.5	18.6	32.6	21.5	34.9	13.5	30.8

* Incomplete data.

**Table 2:** Average and maximum surface temperatures of the partition for the period of July–August.

		July–August 2017 *		July–August 2018		July–August 2016		5 July	
		Coating		Coating		Building			
		*T_av_*	*T_max_*	*T_av_*	*T_max_*	*T_av_*	*T_max_*	*T_av_*	*T_max_*
		°C							
Coating	Grey	21.1	50.8	21.7	52.7	-	-	-	-
	Red	20.6	46.9	21.3	49.6	22.3	54.1	24.5	40.2
	White	19.7	43.7	20.8	49.1	-	-	-	-
Air		21.1	38.8	21.5	34.9	-	19.7	19.3	26.9

* Incomplete data.

**Table 3:** Absorption coefficients for the selected materials based on the values given in [37].

Material Name and Surface Type	Absorption Coefficient *α*
	High-Temperature Radiation
Red ceramic brick	0.70–0.74
Concrete with smooth surface	0.60
Light concrete	0.42
Dark concrete	0.73
Black roofing felt	0.91

## Data Availability

Data is contained within the article.
